# Primary PCI and Mental Health: A 12-Month Follow-Up Study

**DOI:** 10.3390/healthcare11111620

**Published:** 2023-06-01

**Authors:** Dragoș Vulcănescu, Veronica Gheorman, Daniel Cristian Pîrvu, Venera Cristina Dinescu, Victor Gheorman, Ion Udriștoiu, Alina Maria Paraschiv, Marius Gabriel Bunescu, Mihaela Corina Berceanu, Lavinia Gheorman, Sorin Nicolae Dinescu, Romeo Popa, Cristina Florescu, Adrian Mită, Cătălin Mircea Forțofoiu

**Affiliations:** 1 Department of Psychiatry, University of Medicine and Pharmacy of Craiova, 200349 Craiova, Romania; vulcanescudragos@gmail.com (D.V.); victor.gheorman@umfcv.ro (V.G.); ion.udristoiu@umfcv.ro (I.U.); 2 Department of Cardiology, University of Medicine and Pharmacy of Craiova, 200349 Craiova, Romania; paraschivalina30@yahoo.com (A.M.P.); mihaela.berceanu@umfcv.ro (M.C.B.); cristina.t.florescu@umfcv.ro (C.F.); 3 Department of Internal Medicine, University of Medicine and Pharmacy of Craiova, 200349 Craiova, Romania; pirvu_daniel2005@yahoo.com; 4 Department of Health Promotion and Occupational Medicine, University of Medicine and Pharmacy of Craiova, 200349 Craiova, Romania; 5 Occupational Medicine Department, University of Medicine and Pharmacy of Craiova, 200349 Craiova, Romania; marius.bunescu@umfcv.ro; 6 Department of Diabetology, University of Medicine and Pharmacy of Craiova, 200349 Craiova, Romania; lmgheorman@yahoo.com; 7 Department of Epidemiology, University of Medicine and Pharmacy of Craiova, 200349 Craiova, Romania; sorin.dinescu@umfcv.ro; 8 Department of Pharmacology, University of Medicine and Pharmacy of Craiova, 200349 Craiova, Romania; romeo_rop@yahoo.com; 9 Internal Medicine Department, University of Medicine and Pharmacy of Craiova, Filantropia Hospital of Craiova, 200143 Craiova, Romania; adrian.mita@umfcv.ro (A.M.); catalin.fortofoiu@umfcv.ro (C.M.F.)

**Keywords:** PCI, depression, anxiety

## Abstract

The research article highlights the significance of acute myocardial infarction (AMI) and its impact on depression and anxiety among patients’ post-primary percutaneous coronary interventions (PCI). The study aims to determine the frequency of depression and anxiety occurrence in patients with acute myocardial infarction after primary PCI. The objective of this study is to investigate the frequency of depression and anxiety in patients with acute myocardial infarction after primary PCI. The method used in the study involved the collection of data from 88 patients with acute myocardial infarction who underwent primary PCI treatment. The patients were tested before PCI and then at intervals of 1 month, 6 months, and 12 months post-PCI using the Hamilton Depression Scale (HAM-D17) and the Hamilton Anxiety Scale (HAM-A) to identify depression and anxiety symptoms, respectively. The study performed a comprehensive analysis of the collected data to determine the frequency of depression and anxiety occurrence in post-PCI patients. The study found evidence that primary PCI reduces depression and anxiety in patients who have experienced a myocardial infarction. However, mental health issues continue to be a significant psychological concern for patients post-PCI, impacting their lifestyle, self-care, and treatment adherence. The study suggests that healthcare providers should actively screen and manage psychiatric disorders in patients who have suffered from AMI as they are at an increased risk of mental disorders. In conclusion, the study indicates that depression and anxiety are common issues in acute myocardial infarction survivors, and interventions addressing these conditions should be a routine part of care. The study highlights the need for healthcare providers to be aware of the increased risk of mental disorders in individuals who have suffered from AMI. Understanding the impact of anxiety and depression on post-PCI patients is essential for the development of effective interventions that support patients’ recovery.

## 1. Introduction

Acute myocardial infarction (AMI) is a prevalent health issue that can result in a range of psychological outcomes, including depression and anxiety [1,2]. While primary percutaneous coronary intervention (PCI) is a highly effective treatment option for AMI (acute myocardial infarction), its impact on the psychological well-being of patients is not well understood.

After AMI and PCI, psychological outcomes consistently reported in the literature include depression, anxiety, post-traumatic stress disorder (PTSD), and health-related quality of life (HRQoL) issues. A systematic review and meta-analysis published in the International Journal of Environmental Research and Public Health reported that the prevalence of depression among AMI survivors ranged from 10% to 65% and that anxiety was reported by 9% to 48% of patients [3]. Additionally, a study published in the Journal of Cardiovascular Nursing found that patients who underwent PCI experienced high levels of anxiety both before and after the procedure, which can negatively impact their recovery and HRQoL [4]. Furthermore, a review article published in the European Heart Journal stated that PTSD is a common complication following AMI and PCI, affecting up to 1 in 5 patients [5]. Moreover, studies have consistently reported that AMI and PCI can lead to reduced HRQoL, impacting physical functioning, emotional health, and social well-being [6,7]. Hence, effective management of psychological outcomes is critical in the overall management of patients with AMI and PCI.

According to recent studies, there is a growing concern about the psychological outcomes following primary percutaneous coronary intervention (PCI) for acute myocardial infarction (AMI). In our study, we aim to specifically analyze the prevalence of depression and anxiety in patients undergoing primary PCI.

However, there is a wide range of other psychological consequences that can occur after an acute myocardial infarction or PCI procedure, such as:

1. Sleep disorders: difficulty falling asleep, frequent waking during the night, restless sleep.
2. Post-traumatic stress: symptoms such as nightmares, flashbacks, avoidance of certain situations, hypervigilance, etc.
3. Anger and irritability: symptoms such as aggression, irritability, uncontrolled anger.
4. Decreased self-confidence: feelings of worthlessness, hopelessness, decreased sexual appetite, lack of satisfaction with activities that used to bring joy, etc.
5. Concentration problems: difficulties in multitasking, decision-making or maintaining focus on a task, etc.

It is important to understand that all of these repercussions can have a significant impact on quality of life after AMI or PCI. Proper diagnosis and treatment of psychological problems associated with coronary artery disease is necessary.

Conditions such as sleep disorders, post-traumatic stress, and irritability can significantly impact a person’s quality of life after an acute myocardial infarction or PCI.

Sleep disorders such as insomnia, sleep apnea, and restless leg syndrome can disrupt a person’s ability to get sufficient sleep, which can lead to fatigue, lack of energy, and difficulty concentrating. This can affect their ability to engage in daily activities, including exercise, work, and socializing.

Post-traumatic stress disorder (PTSD) is a mental health condition that can occur after a person experiences a traumatic event, such as an acute myocardial infarction or PCI. Symptoms of PTSD can include flashbacks, nightmares, and avoidance behaviors, which can interfere with a person’s ability to function and enjoy life.

Irritability is a common symptom after an acute myocardial infarction or PCI and can impact a person’s relationships with family members, friends, and coworkers. It can also lead to feelings of frustration, anger, and depression, which can negatively impact a person’s mental health and overall well-being.

In short, sleep disorders, post-traumatic stress and irritability can all have a significant impact on a person’s quality of life after an acute myocardial infarction or PCI. It is important for healthcare providers to address these conditions and offer treatment options to improve a person’s physical and mental health.

The impacts of sleep disturbances, decreased self-confidence, depression, and anxiety on the quality of life of acute myocardial infarction (AMI) survivors and percutaneous coronary intervention (PCI) patients were examined in recent studies. The Journal of Clinical Sleep Medicine published a study in 2020 that found sleep disorders, such as insomnia and sleep apnea, were associated with a lower quality of life and increased risk of depression and anxiety in AMI survivors [8]. The Journal of Cardiology published another study in 2020, which found that decreased self-confidence was negatively associated with quality of life in PCI patients [9]. Furthermore, a systematic review and meta-analysis published in the Journal of Affective Disorders in 2020 found that depression and anxiety were significant predictors of poor health-related quality of life in AMI patients [10].

A study by Pedersen, Denollet and Spinder [11] aimed to investigate the prevalence and severity of depression among patients who had recently experienced an acute myocardial infarction (AMI), as well as to identify potential predictors of these outcomes [11]. The researchers collected data from 294 patients who had been hospitalized with an AMI and were undergoing percutaneous coronary interventions (PCI). The patients completed self-report questionnaires that measured depression, anxiety, physical limitation, and social support at baseline and one week after discharge. The specific objectives of the study were to determine the prevalence and severity of depression and anxiety symptoms in patients with AMI one week after discharge, to identify potential predictors of depression and anxiety symptoms in patients with AMI one week after discharge, and to explore the relationship between depression and anxiety symptoms, physical limitation, and social support in patients with AMI. The study found that approximately one-third of patients experienced clinically significant symptoms of depression and/or anxiety in the week after their AMI. The researcher also identified several factors that were associated with higher levels of depression and anxiety, including younger age, female gender, low social support, and physical limitations.

Research has shown that the prevalence of psychiatric disorders among individuals affected by cardiovascular disease is approximately 2–3 times higher compared to those without such conditions [12,13,14,15].

According to a study by Farooq et al., patients with coronary artery disease (CAD) have a significantly higher prevalence of psychiatric disorders than the general population [16]. The researchers conducted a cross-sectional study of 384 patients with CAD who were attending outpatient clinics in a tertiary care hospital in Pakistan. The patients were evaluated using the Mini International Neuropsychiatric Interview (MINI). The specific objectives of the study were to: determine the prevalence of psychiatric disorders among patients with CAD and to identify the most common psychiatric disorders among patients with CAD. The researchers analyzed the data using descriptive statistics to determine the prevalence of psychiatric disorders and logistic regression to identify factors associated with the presence of psychiatric disorders. The researchers found that over half (54.2%) of patients with CAD had some form of psychiatric disorder. This is a much higher prevalence than the range observed in the general population, which is typically between 7 and 20%.

Rani et al. performed a cross-sectional study at Sir Sunderlal Hospital, Banaras Hindu University, India [17]. The study included 152 patients who underwent primary PCI for acute ST-elevation myocardial infarction (STEMI) between January 2018 and December 2018. Depression was assessed using the Patient Health Questionnaire-9 (PHQ-9). Their objective was to determine the prevalence of depression among patients undergoing primary PCI for STEMI. This study found that depression was common among patients undergoing primary PCI, with an overall prevalence of 58%.

Wang et al., (2020) realized a retrospective cohort study conducted in a single center in China [18]. The study included 463 patients who underwent primary PCI for acute myocardial infarction (AMI) between January 2016 and December 2018. Anxiety was assessed using the Hospital Anxiety and Depression Scale (HADS) at admission. The primary outcome was major adverse cardiovascular events (MACE) at 12 months. Their objective was to investigate the association between anxiety and prognosis in patients undergoing primary PCI for AMI. This study found that anxiety was independently associated with worse prognosis in patients undergoing primary PCI for AMI.

Liu et al., (2020) conducted a cross-sectional study on 284 patients who underwent primary percutaneous coronary intervention (PCI) for acute myocardial infarction (AMI) [19]. The Patient Health Questionnaire-9 (PHQ-9) was used to assess depression, and the Short Form-36 (SF-36) was used to assess the quality of life. Logistic regression analyses were performed to determine the association between depression and quality of life in these patients. The objective of this study was to investigate the association between depression and quality of life in patients undergoing primary PCI for AMI. This study found that depression was associated with worse quality of life in patients undergoing primary PCI for AMI.

Liu et al., (2021) performed a systematic review and meta-analysis of 13 observational studies involving 5738 patients who underwent primary PCI for acute coronary syndrome (ACS) [20]. The studies were selected from five electronic databases, resulting in a total of 10 cohort studies and 3 case-control studies. The quality of the studies was assessed using the Newcastle-Ottawa Scale, and the data were analyzed using a random effects model to calculate pooled hazard ratios. The objective of this study was to evaluate the association between depression and major adverse cardiovascular events (MACE) in patients undergoing primary PCI for ACS. This study found that depression was independently associated with an increased risk of major adverse cardiovascular events in patients undergoing primary PCI.

Li et al. conducted a cross-sectional study to investigate the prevalence of anxiety and depression in patients with acute myocardial infarction (AMI) undergoing primary percutaneous coronary intervention (PCI) [21]. The study recruited 180 patients from a single center in China between October 2018 and December 2019. The participants completed the Hospital Anxiety and Depression Scale (HADS) to assess their levels of anxiety and depression. The researchers found that 51.7% of the patients had anxiety symptoms, 32.2% had depression symptoms, and 25.6% had symptoms of both anxiety and depression. Additionally, the study found that anxiety and depression were associated with worse health outcomes and increased length of hospital stay.

Karthikeyan et al. conducted a cross-sectional study to investigate the impact of social support on anxiety and depression in patients undergoing primary PCI for AMI [22]. The study recruited 150 patients from a tertiary care hospital in India between January 2019 and December 2019. The participants completed the HADS and the Multidimensional Scale of Perceived Social Support (MSPSS) to assess their levels of anxiety, depression, and perceived social support. The researchers found that perceived social support was associated with lower levels of anxiety and depression in patients undergoing primary PCI for AMI. The study suggests that providing social support may be a potential strategy to improve mental health outcomes in this patient population.

Within this context, it is imperative to conduct screening at every visit (or 2–4 times annually). The 12-month prevalence of mental disorders among patients with cardiovascular disease is approximately 40% leading to a significantly worse prognosis [23,24,25,26]. The onset of cardiovascular disease heightens the risk of suicide. In light of this, there should be an increased awareness of anxiety and depression symptoms.

The treatment of acute myocardial infarction has improved significantly with the widespread use of primary percutaneous coronary intervention (PCI). However, little is known about the long-term impact of this invasive procedure on the psychological well-being of patients. As such, the present study aims to answer the research question: “Does undergoing primary PCI for acute myocardial infarction have an impact on a patient’s mental health in the long term?” This question will guide our research and analysis of the 12-month follow-up data, allowing us to explore the potential effects of primary PCI on mental health outcomes and identify areas for future research in this field.

In this article, we emphasize the significance of our study in terms of the multitude of measures taken throughout the course of the year. This allows us the opportunity to diligently monitor and analyze trends over time.

## 2. Materials and Methods

The article examined the published research document and performed a comprehensive analysis of our collected data to ascertain the frequency of anxiety and depression occurrence among patients post-primary PCI.

The study protocol was approved by the Ethics Committee and is in accordance with the Helsinki Declaration of 1975. All subjects gave their informed consent for inclusion before participating in the study. In our research, the process involved explaining the study’s purpose, procedures, potential risks, and benefits, and emphasized voluntary participation. We made sure that participants fully understood the information provided, their rights in the study, and the confidentiality measures. Informed consent involved verbal discussions and electronic consent forms.

The study utilized a longitudinal design with four-time points (before PCI, 1 month, 6 months, and 12 months post-PCI), allowing for the evaluation of changes in anxiety and depression levels over time. The use of data collected at multiple time points increases the reliability and validity of the findings.

The selection criteria were determined based on the research question and the population of interest.

The selection criteria used in this study included factors such as age, gender, medical history, and specific symptoms or conditions (acute myocardial infarction).

In our study, the sample size of 88 was determined based on the research question, available resources, and the expected effect size of the intervention being studied.

To assess the representativeness of the sample, we considered the demographic characteristics of the sample (age, sex and environment), as well as other important factors that may influence the results (health status).

The study examined patients after an acute myocardial infarction with a variety of medical conditions, such as hypertension and diabetes mellitus, and the patients’ risk factors such as smoking, alcohol, and hypercholesterolemia (Table 1).

Exclusion criteria included a history of acute myocardial infarction, age under 18 years and over 80 years, inability to complete the test, inability to complete the 12-month monitoring, and severe chronic illness with a poor prognosis (malignancy, stroke). However, 19 subjects from our sample (107 subjects in total) were excluded due to severe chronic illness with a poor prognosis.

To identify depression and anxiety, we chose to use standardized measures such as the Hamilton Depression Scale (HAM-D17). Compared to other tests that evaluate depressive symptomatology, HAM-D17 has the advantage of evaluating non-cognitive elements that other scales usually do not consider [27].

The Hamilton Depression Scale (HAM-D17) consists of 17 items that reflect a depressive mood, feelings of guilt, suicidal ideation or behavior, insomnia, difficulties in professional and somatic functioning, gastrointestinal symptoms, general somatic symptoms, genital symptoms, hypochondria, weight loss, and insight into the condition.

The final score is obtained by adding up the scores of all the items and ranges from 0 to 54, with depression severity being assessed differently: 0–7 indicates the absence of depression, 8–14 indicates mild depression, 15–22 indicates moderate depression, and greater than 23 indicates severe depression with suicidal risk.

Anxiety symptoms were evaluated using the Hamilton Anxiety Scale (HARS or HAM-A). This scale was developed in 1959 to quantify the severity of anxiety and does not measure a specific clinical entity but provides a global assessment of anxiety [28].

The scale contains 14 items that measure both anxiety (anxious mood, fear, difficulty concentrating, psychological tension, depressive mood) and somatic symptoms (insomnia, muscle tension, sensory changes, gastrointestinal, cardiovascular, genitourinary, respiratory, and vegetative).

Each item is rated on a 5-point scale from 0 (absence of anxious symptoms) to 4 (severe, disabling symptoms). The score obtained by adding up the responses to the items ranges from 0 to 56, and the severity of symptoms is estimated as follows: 0–17 indicates mild anxiety, 18–24 indicates moderate anxiety, and 26–30 indicates severe anxiety.

The Hamilton Depression Rating Scale-17 (HAM-D17) and Hamilton Anxiety Rating Scale (HAM-A) are widely used tools to evaluate the severity of depressive and anxiety symptoms, respectively. They are considered reliable and valid measures of mood and anxiety symptoms in clinical populations dealing with depression and/or anxiety.

Concerning their reliability, both HAM-D17 and HAM-A are widely used and have high levels of internal consistency and test-retest reliability. The HAM-D17 has been proven to have good inter-rater reliability with correlation coefficients ranging from 0.80 to 0.94. The HAM-A also has good inter-rater and test-retest reliability, with inter-rater reliability coefficients ranging from 0.40 to 0.90.

Regarding their validity, both scales have a good level of content validity. This means that the items on the scale measure the intended construct effectively. Additionally, HAM-D17 and HAM-A have good convergent and discriminant validity, which means that the scale correlates with other measures of the same construct and does not correlate with measures of different constructs. For example, HAM-D17 correlates strongly with the Beck Depression Inventory (BDI), and weakly with measures of anxiety. Similarly, HAM-A correlates strongly with the State-Trait Anxiety Inventory, and weakly with measures of depression.

Both the HAM-D17 and HAM-A have been widely utilized in studies involving patients who experienced an acute myocardial infarction (AMI) and received primary PCI. A literature review shows that these scores significantly increase in patients with AMI compared to the general population, and subsequently decrease over time with proper treatment. Furthermore, both scales have been used to evaluate the effectiveness of various treatments for depression and anxiety in patients with AMI, including pharmacological and psychotherapeutic interventions. For example, a study published in the Journal of the American Heart Association examined depression and anxiety in 207 patients who underwent primary PCI for AMI [29]. The authors discovered that higher levels of depression and anxiety were related to a poorer recovery trajectory and more adverse outcomes after discharge. Another study published in the Journal of Psychosomatic Research used the HAM-D17 to assess depressive symptoms in 242 patients with AMI who received primary PCI [30]. The authors determined that higher levels of depression were correlated with a lower quality of life and increased mortality risk during follow-up.

While the HAM-D17 and HAM-A are extensively used and validated measures of symptoms for depression and anxiety, limitations and considerations must be taken into account. For instance, the scales may not be sensitive to all forms of depression or anxiety and may not be suitable for use in patients with severe cognitive impairment. Furthermore, the scales rely on patients’ self-reported symptoms and do not directly measure the physiological or biological correlates of depression and anxiety, which can be influenced by biases and inaccuracies. Additionally, these scales may not fully cover all depression and anxiety symptoms experienced by patients and may not detect minor or nuanced changes in symptoms over time. Finally, the scores on the scales can be influenced by other factors such as physical health, medications, and social support, which should be considered when interpreting the results.

In our research examining depression and anxiety after an acute myocardial infarction (AMI) treated via primary percutaneous coronary intervention (PCI), we collected data on various variables and used specific statistical methods. We compared and correlated the HAM-D17 and HAM-A scales to evaluate depression and anxiety symptoms in patients with AMI treated via primary PCI. We assessed the relationship between depression and anxiety symptoms and clinical factors such as age, gender, and medical history. Additionally, we evaluated the relationship between depression and anxiety symptoms, as measured by the HAM-D17 or HAM-A, at four time points (before PCI, 1 month, 6 months, and 12 months post-PCI). We also collected data on psychiatric disorders (depression, anxiety) and family history of mental health and other medical conditions, as well as information on antidepressant medication use.

Information on psychiatric disorders was collected through diagnostic interviews and self-reported questionnaires completed by patients, with the option for follow-up interviews to clarify responses. The patients were interviewed by psychiatrists.

Family history information was obtained through interviews with patients and through medical records. A structured family history questionnaire was used to obtain standardized information on the family history of mental health and medical conditions.

Information on antidepressant medication use was obtained through medical records, medication lists provided by patients, and self-reported questionnaires.

Medical conditions were assessed through self-report by patients, review of medical records, and physical examinations. In all cases, specialists were consulted to confirm diagnoses.

The statistical methods we used to analyze these relationships were based on our research question, specifically whether undergoing primary PCI for acute myocardial infarction has an impact on a patient’s long-term mental health. To effectively analyze this question, we utilized correlative analyses (Pearson’s correlation coefficient) to examine the relationship between depression or anxiety symptoms and outcomes of interest.

To calculate confidence intervals for correlation coefficients, we used Fisher’s Z transformation to convert the correlation coefficient to a normal distribution, which allowed for the calculation of confidence intervals using standard methods. Fisher’s Z transformation involves applying the inverse hyperbolic tangent function to the correlation coefficient, resulting in a distribution that is approximately normal. Confidence intervals were then calculated using the standard formula for the mean and variance of a normal distribution.

In addition to correlative and regression analyses, we also utilized *t*-tests to compare means between groups. For example, throughout our study, we used *t*-tests to compare the mean scores of anxiety symptoms between patients before primary PCI, at 1 month, 6 months, and 12 months. This allowed us to determine if there was a statistically significant difference in anxiety levels. The resulting *t*-value was compared to a critical value to determine if the difference was statistically significant. Overall, these statistical methods helped us to robustly analyze and interpret the data, providing valuable insight into the impact of primary PCI on mental health outcomes.

The data analysis methods were conducted using MedCalc 15.6.4 and MS Office Excel 2007. MedCalc was utilized for performing statistical analyses, such as descriptive statistics, correlative analyses (Pearson’s correlation coefficient), and *t*-tests. MS Office Excel 2007 was employed to create graphs and charts to display the study’s results.

## 3. Main Findings of This Work

The research included 88 subjects with acute myocardial infarction who underwent PCI (65 men and 23 women). The mean age of the cohort was 65.74 ± 7.54 years (range 46–78 years), being higher in women (70.22 ± 5.08) than in men (64.15 ± 7.66).

### 3.1. Depression

The pre-intervention Hamilton D 17 score was 7.59 ± 2.05, indicating a mild depressive state, which was over 40% higher than the score measured one month after the intervention (*p* < 0.001) (Table 2). Almost half of the included subjects fell into the category of mild depression (52.3%, N = 46).

After the intervention, there was a significant reduction in the level of depression, as evidenced by the mean Hamilton score for depression one month later being reduced to 5.32 ± 2.68 (Table 2). Only 16 subjects (18.2%) still fell into the category of mild depression, which is nearly threefold lesser in comparison to the pre-intervention phase. The level of depression remained low at six-month mark (4.06 ± 2.45), with 11 subjects exhibiting mild depression (12.5%). This trend persisted at the twelve-month mark (4.35 ± 2.54) (Table 2).

The results of our study suggest that primary PCI may have a positive impact on reducing depression in patients who have experienced a myocardial infarction. This is consistent with previous studies that have shown a significant reduction in depression following cardiac intervention such as PCI [31,32,33].

Consistent with other recent research, studies have found that depression levels decreased significantly in the first 3 months after PCI, but increased thereafter, with a higher rate of depression recurrence in patients with a history of depression [34,35,36].

These studies suggest that depression levels tend to improve after PCI, but can increase again over time, as observed in our study. It is important to continue monitoring depression levels in patients undergoing PCI and to provide appropriate follow-up treatment as needed.

Furthermore, the reduction in depression observed in our study may have implications for long-term survival. A recent meta-analysis found that depression is a significant predictor of mortality following myocardial infarction, with depressed patients having a 2.03 times greater risk of mortality compared to non-depressed patients [37].

These findings highlight the importance of addressing mental health concerns in patients with cardiovascular disease to optimize overall health outcomes.

#### 3.1.1. Gender

Women presented a higher Hamilton score for depression than men, regardless of the questionnaire completion time. Before the intervention, women had a HAM-D17 score of 8.78 ± 2.04 compared to men’s 7.17 ± 2.53 (*p* < 0.001), with almost three-quarters of women (73.01%, N = 6) presenting with mild depression compared to only 44.62% (N = 29) in men (*p* = 0.03) (Table 2).

In a study by Koivula M. et al., the authors examine depressive symptoms in a cohort of 276 patients undergoing PCI. They found that women had significantly higher depression scores at baseline and at 6 months after PCI compared to men [38].

These findings highlight the importance of screening for depressive symptoms in women undergoing PCI and proving appropriate intervention and support. So, we suggest that:

1. Early detection and treatment of depression in post-PCI women is crucial
2. Integrating mental support into cardiac rehabilitation programs could be beneficial for women post-PCI
3. Identifying women who are at a higher risk of depression post-PCI could help healthcare providers tailor their care to support these individuals
4. Implementing routine screening for depression in women who have undergone PCI could help prevent and/or manage depressive symptoms.

**Figure 1:**
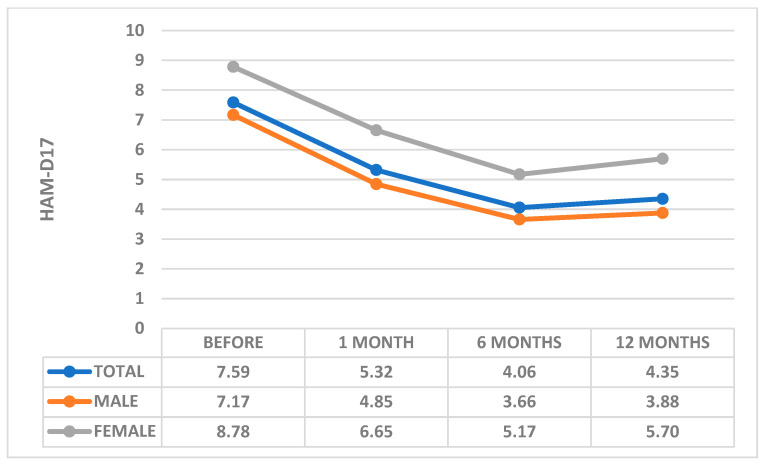
Comparative Analysis of Depression Severity by Gender. HAM-D17: Hamilton Depression Rating Scale-17.

Both sexes recorded a significant decrease in HAM-D17 score after the first month following the intervention, more pronounced (*p* = 0.004) in men (4.85 ± 2.59) than in women (6.65 ± 2.53) (Figure 1).

The decrease continued and stabilized at 6 months after the intervention, with the HAM-D17 score in women (5.17 ± 2.5) and men (3.66 ± 2.32) showing a decrease of 70% and 95%, respectively, compared to the values before PCI intervention (Figure 1).

The slight increase in the HAM-D17 score recorded at 12 months after PCI intervention (5.7 ± 1.96 in women and 3.88 ± 2.56 in men) was not significant, both in women (*p* = 0.45) and in men (*p* = 0.61) (Figure 1).

Depression following myocardial infarction is a frequent and serious comorbidity, with prevalence rates ranging from 14–47%. Studies suggest that the highest rates of depression occur in the first six months post-MI, with some evidence of subsiding after one year. Female patients, those with a more severe acute coronary syndrome (ACS) are at higher risk for post-MI depression [39,40].

#### 3.1.2. Age

After one month from PCI intervention, the decrease in the HAM-D17 score was more significant for subjects under 60 years. The highest HAM-D17 score before the intervention was found in subjects over 70 years old (9.2 ± 1.98) and the lowest in those under 60 years old (9.2 ± 1.98). One year after PCI, subjects over 70 years old had HAM-D17 scores (6.84 ± 2.59) almost double (*p* < 0.001) compared to those under 60 years old (3.38 ± 1.54) (Table 2).

The results indicate that there are differences in HAM-D17 scores between age groups before and after PCI. Patients below 60 years of age had lower HAM-D17 scores before PCI compared to patients in the other age groups. However, all age groups showed a decrease in HAM-D17 scores after PCI. Patients in the 60–70 age group had the highest HAM-D17 score before PCI and showed the greatest improvement in scores 6 months after PCI. Patients above 70 years of age had the highest HAM-D17 scores before and after PCI. Additionally, this group showed the smallest improvement in HAM-D17 scores over time. These findings suggest that age may play a role in the level of depression experienced by patients after myocardial infarction and that older patients may require more targeted and ongoing support to manage these symptoms.

Healthcare providers should be aware of the prevalence of anxiety and depression in elderly patients, especially those who have undergone cardiovascular procedures. It is important to recognize the unique challenges that elderly patients face, such as social isolation and reduced mobility, when dealing with post-procedure psychological concerns. Untreated psychological issues can have a negative impact on the patient’s overall well-being and adherence to treatment plans. Therefore, healthcare providers should prioritize the assessment and treatment of psychological concerns in elderly patients, as this greatly improves their quality of life and overall health outcomes.

#### 3.1.3. Co-Existing Conditions

The results of our study suggest that patients with hypertension may experience higher levels of anxiety and depression after percutaneous coronary intervention (PCI) compared to those without hypertension. This is indicated by the higher HAM-D17 scores observed in hypertensive patients before and after PCI, as compared to non-hypertensive patients (Table 2).

This finding is in line with previous studies that have also reported higher levels of psychological distress among patients with cardiovascular diseases and hypertension. For example, a systematic review and meta-analysis by Mancia et al. found that hypertension was associated with a higher risk of depression in patients with cardiovascular diseases [41].

Our study also suggests that over time, both hypertensive and non-hypertensive patients experienced improvements in their HAM-D17 scores after PCI. This is consistent with previous research showing that PCI can have positive effects on psychological outcomes in patients with cardiovascular disease [42].

Additionally, the results of our study suggest that patients, both with and without diabetes mellitus, who undergo percutaneous coronary intervention (PCI) for myocardial infarction have a significant reduction in anxiety and depression symptoms post-procedure. The HAM-D17 scores for both groups decreased significantly at 1 month, 6 months, and 12 months after the procedure.

A study by Hassan et al. found that patients who underwent PCI had a significant reduction in depressive symptoms as early as 1-week post-procedure and up to 1 year later [43]. The study also found that patients with diabetes had higher baseline depressive symptoms than those without diabetes, but there were no significanct differences in depressive symptoms between the two groups post-PCI. Similarly, a study by Wu et al. found that PCI significantly reduced depressive symptoms in patients with acute coronary syndrome [44]. The study also found that patients with diabetes had higher baseline depressive symptoms than those without diabetes, but there were no significant differences in depressive symptoms between the two groups post-PCI.

Overall, our study and previous research suggest that PCI can significantly reduce anxiety and depression symptoms in patients with and without diabetes mellitus.

Healthcare professionals must provide comprehensive care to address the physical and emotional needs of patients, including monitoring their blood pressure and blood sugar levels, and providing mental health support. By taking a holistic approach to care, patients can have a better chance of achieving successful outcomes and returning to healthy, fulfilling lives.

**Figure 2:**
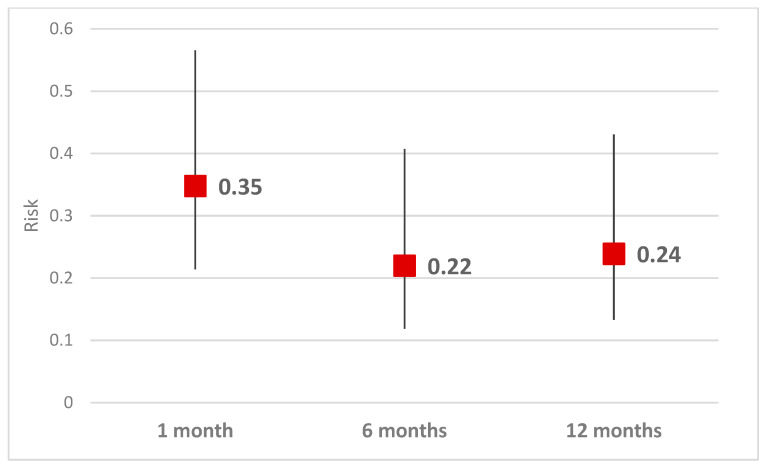
Risk of Depresion Comparing with Preintervention Level.

The risk of depression (HAM score > 7) decreased strongly in the first month after the intervention (RR = 0.35, CI95% 0.22–0.57, *p* < 0.001), continued to decrease at 6 months (RR = 0.22, IC95% 0.12–0.41, *p* < 0.001), and stabilized at 12 months (Table 3, Figure 2).

Research has shown that patients who undergo percutaneous coronary intervention (PCI) for the treatment of acute myocardial infarction (AMI) are at an increased risk of developing depression. However, studies have also suggested that timely intervention and treatment can help alleviate depression symptoms in these patients.

According to a study conducted by Lesperance and colleagues, the risk of depression decreases significantly in patients with AMI who undergo PCI, particularly within the first few months post-intervention [45]. The study found that the risk of depression decreased strongly in the first month after the intervention (*p* < 0.001), continued to decrease at 6 months (*p* < 0.001), and stabilized at 12 months.

Taken together with our findings suggest that early intervention and treatment of depression in patients with AMI who undergo PCI can result in significant improvements in mental health outcomes. Additionally, it highlights the importance of ongoing monitoring and support for patients in the months following PCI to ensure that they continue to receive the necessary care and treatment to manage depression symptoms.

As a result, it is important for healthcare providers to not only focus on the physical health of patients undergoing PCI, but also on their mental health and well-being. This can be achieved through the implementation of comprehensive care plans that encompass both physical and mental health interventions.

Depression is a common psychological condition that often develops after myocardial infarction (MI). MI patients who develop depression experience a range of negative outcomes, including increased risk of mortality, heightened cardiovascular morbidity, reduced quality of life, and increased healthcare utilization [46].

### 3.2. Anxiety

Anxiety after acute myocardial infarction (AMI) treated by percutaneous coronary intervention (PCI) is a significant psychological concern for patients. While PCI is an effective treatment for AMI, it is not without its challenges. Patients may experience physical discomfort, fear of death, and feelings of vulnerability during and after the procedure. Anxiety in post-PCI patients can impact their lifestyle, self-care, and treatment adherence.

Understanding the impact of anxiety on post-PCI patients is essential for the development of effective interventions that support patient recovery.

Based on our study, we found that the mean HAM-A score before PCI was 7.227, indicating high levels of anxiety among patients with acute myocardial infarction. However, we observed a significant decrease in anxiety levels at 1-month post-PCI, with mean scores dropping to 4.67. This reduction was further sustained at 6 months, with mean HAM-A scores decreasing to 2.989. Interestingly, we observed a slight increase in anxiety levels at 12 months, with mean HAM-A scores rising to 3.545. Our findings are consistent with previous studies that have reported reductions in anxiety levels following PCI [47]. However, some studies have also reported a gradual increase in anxiety over time, which may explain the slight increase we observed at the 12-month follow-up [48,49]. It is possible that patients may experience lingering concerns about their cardiac health or adjust to a new baseline level of anxiety following the initial relief post-procedure.

Overall, our study adds to the growing body of evidence pointing to the positive impact of PCI on anxiety levels among patients with acute myocardial infarction. However, further research is needed to better understand the long-term effects of these interventions on psychological well-being and to identify strategies for mitigating anxiety over time.

**Figure 3:**
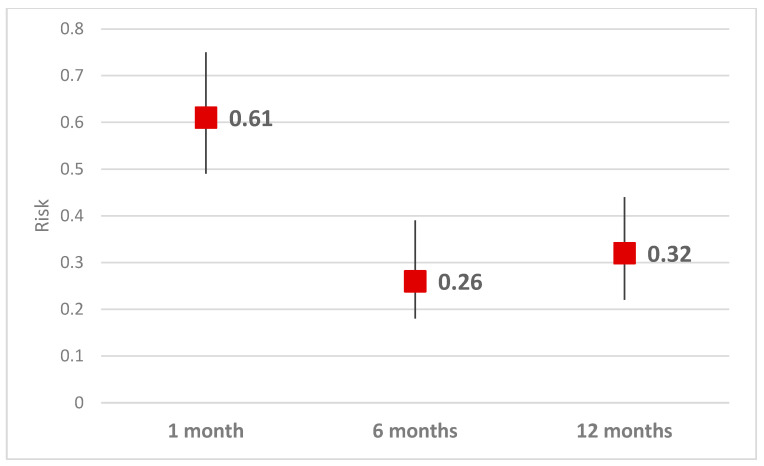
Risk of Anxiety Comparing With Preintervention Level.

The risk of anxiety compared with pre-interventional level, calculated for HAM score higher than four (including subjects with slight and moderate anxiety), decreased in the first month after the intervention (RR = 0.61, CI 95% 0.49–0.75, *p* < 0.001), and at 6 months it reached the lowest level (0.26, CI 95% 0.18–0.39, *p* < 0.001). At 12 months, the risk of anxiety stabilized at 0.32 compared to the anxiety before the intervention (RR = 0.32, CI 95% 0.22–0.44, *p* < 0.001) (Table 4, Figure 3).

Our results suggest that the risk of anxiety decreases significantly after PCI intervention, with the lowest point being at 6 months post-intervention and stabilizing at 12 months. These findings are consistent with several other studies that have explored the link between interventions for cardiovascular disease and anxiety [49]. One study by Korhonen et al. found that cardiac rehabilitation significantly reduced anxiety symptoms and improved overall psychological symptoms and improved overall psychological well-being in patients with cardiovascular disease [50]. Another study by Ostvar et al. found that cognitive behavioral therapy was an effective intervention for reducing anxiety in patients with heart disease [51].

Overall, these findings suggest that interventions aimed at treating cardiovascular disease can also have a positive impact on anxiety symptoms, providing important implications for the care of patients with these conditions.

#### 3.2.1. Gender

The result of our study suggest that percutaneous coronary intervention (PCI) may have a positive impact on anxiety levels in both male and female patients following myocardial infarction. The HAM-A scores decreased significantly for both genders at each time point, indicating a gradual improvement in anxiety levels over the course of the study (Table 5). While both groups experienced a decrease in HAM-A score post-PCI, female patients had higher scores both before and after the procedure (Table 5).

Research has shown that women may be more succeptible to anxiety and depression following a cardiac event, possibly due to differences in physiological responses and social factors [52,53,54].

We concluded that in our study are gender differences in anxiety among MI patients treated with PCI. We highlight the importance of identifying and managing anxiety in female MI patients and suggest that gender-specific interventions should be developed to optimize patients outcomes following PCI.

#### 3.2.2. Age

**Figure 4:**
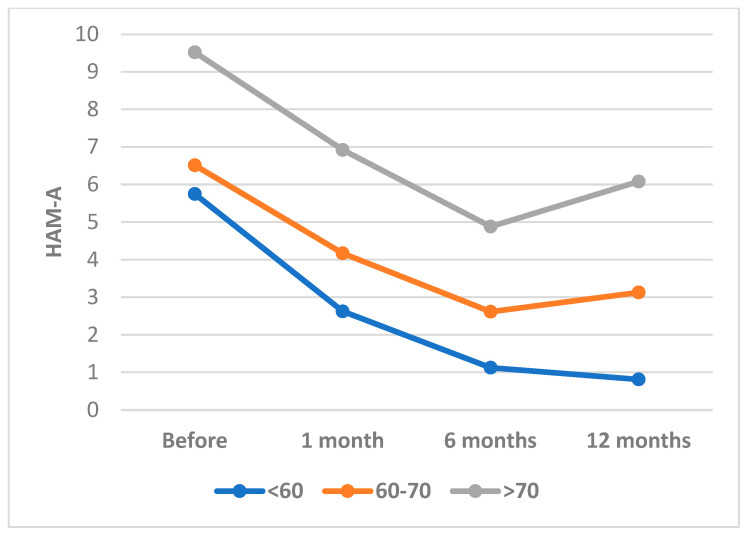
HAM-A Scores Over Time by Age Category. HAM-A: Hamilton Anxiety Scale.

**Figure 5:**
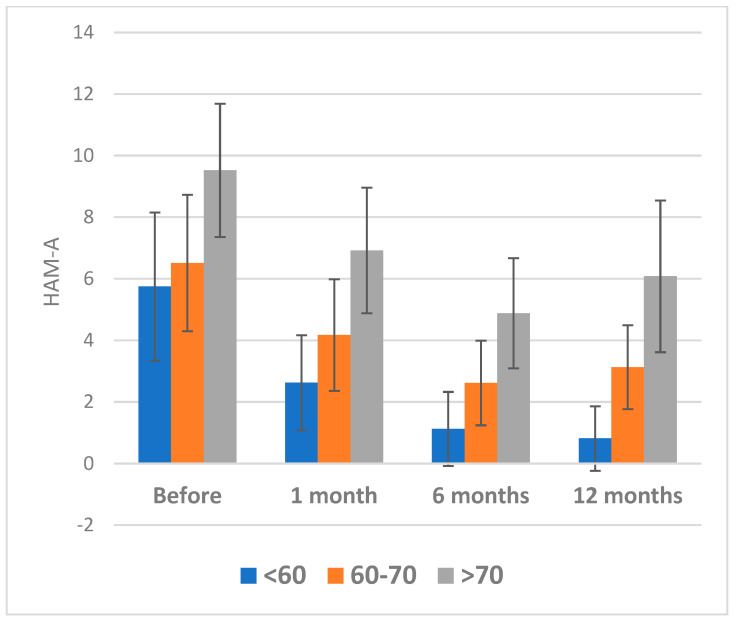
Changes in HAM-A Scores Before and After PCI by Age Group. HAM-A: Hamilton Anxiety Scale.

The results of or study indicate that patients who have undergo percutaneous coronary intervention (PCI) for myocardial infarction experience a significant reduction in symptoms of anxiety over time (Figure 4, Figure 5). This is supported by a statistically significant decrease in HAM-A scores across all age groups at each follow-up time point.

Several previous studies have also identified a reduction in anxiety symptoms following PCI [55,56]. Another study found that patients who receive a combination of cognitive-behavioral therapy and standard care after PCI had significantly reduced anxiety and depression symptoms compared to those who received standard care alone [57].

However, it should be noted that older patients (>70 years) in this study had higher baseline HAM-A scores and a slower rate of improvement compared to younger patients, which is consistent with previous research indicating that older age is a risk factor for worse mental health outcomes following cardiac events [58,59] (Figure 5).

Therefore, interventions to address mental health concerns in older patients may need to be tailored to their unique needs and circumstances. We suggest that healthcare providers should consider the higher risk of anxiety in older patients undergoing PCI and provide appropriate psychological support to these patients.

#### 3.2.3. Comorbidities

The results of this study suggest that patients who undergo percutaneous coronary intervention (PCI) for myocardial infarction experience a significant improvement in anxiety and depression symptoms over time (Table 5).

Both patients with and without hypertension experienced a similar pattern of improvement, with decreasing scores on the HAM-A scale at 1 month, 6 months, and 12 months after PCI compared to baseline.

Previous research supports these findings, with studies showing that PCI can lead to improvements in mental health outcomes such as anxiety and depression [60]. For example, a meta-analysis of 31 studies found that PCI was associated with reduced symptoms of anxiety and depression at both short- and long-term follow-up periods.

Importantly, this study highlights the need to consider comorbidities such as hypertension when assessing the impact of PCI on mental health outcomes. While patients with and without hypertension both experienced improvement, there were differences in the magnitude of change over time.

In terms of diabetes mellitus, the improvement appears to be greater in patients with diabetes mellitus than in those without. Research supports the association between diabetes mellitus and increased risk of anxiety and depression. A study by Talbot et al. found that patients with diabetes mellitus are more likely to experience anxiety and depression than those without diabetes mellitus [61]. This may be due to the chronic stress associated with managing diabetes mellitus, as well as the possible physiological effects of the disease on mood regulation.

**Figure 6:**
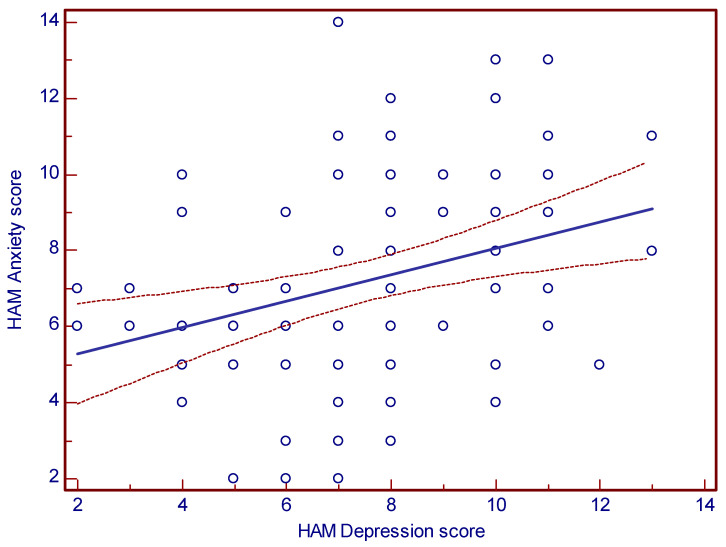
Relationship between Depression and Anxiety Scores on Hamilton Scale.

The correlation between the two Hamilton scores for depression and anxiety was important (r = 0.33, CI 95% 0.12–0.5, *p* = 0.002), suggesting that the two conditions support each other (Figure 6).

Our findings of a significant positive correlation between Hamilton scores for depression and anxiety in patients after an AMI treated by PCI are consistent with existing literature [62].

A review article by Lichtman et al. summarized several studies that found a significant association between depression and anxiety in post-AMI patients [63]. For example, a study by Frasure-Smith et al. reported a correlation coefficient of 0.44 between depression and anxiety scores in post-AMI patients, while a study by Farrell et al. found a significant positive correlation (r = 0.43) between anxiety and depression in patients who underwent PCI [42,64].

Furthermore, a meta-analysis by Celano et al. found that co-occurrence of depression and anxiety symptoms is common in post-AMI patients and is associated with worse outcomes, such as increased mortality and reduced quality of life [65].

Given these findings, it is important to assess and treat both depression and anxiety in post-AMI patients to improve their overall well-being and prognosis.

In conclusion, anxiety is a common emotion experienced by patients undergoing percutaneous coronary intervention. Healthcare providers can help alleviate anxiety by proving clear information about the procedure, offering support and reassurance, and answering any questions or concerns that patients may have. Addressing anxiety before and after the procedure can lead to improved patient outcomes and a more positive patient experience.

Additionally, the Hamilton Anxiety Score is a valuable tool to assess anxiety levels in patients undergoing PCI. Identifying patients’ anxiety levels before the procedure can predict outcomes and help healthcare providers adequately manage patients. Psychological interventions can improve anxiety levels and lead to improved outcomes following PCI.

## 4. Limitations

While our study provides valuable insights into the topic at hand, it is important to acknowledge its limitations.

Firstly, the study was conducted using a relatively small sample size, which may limit the generalizability of our findings to the broader population.

Secondly, our sample was drawn from a specific geographical region, which may limit the generalizability of our findings to other regions or cultures.

Additionally, our study relied on self-report measures, which may be subject to participant bias, and we did not include any objective measures of the variables being studied.

Another limitation is the potential for selection bias, as patients who were unwilling or unable to participate may have had different psychological profiles than those who did participate.

In conclusion, while this study provides valuable insights into the frequency and progression of anxiety and depression symptoms post-PCI, further research using larger and more diverse samples, including both self-report and objective measures and incorporating a longitudinal design, is needed to fully understand the psychological impact of PCI on patients.

## 5. Future Lines of Research

Overall, there are many potential future studies on this topic that could provide valuable insights into the impact of primary PCI on mental health in the long term:

1. Conduct a larger, multi-center study: While our research provides valuable insights into the topic, a larger study involving multiple centers and a larger patient population would provide a more comprehensive picture of the impact of primary PCI on mental health in the long term.
2. Long-term follow-up studies: While our study covered a 12-month follow-up period, many patients may continue to experience mental health issues beyond this timeframe. Conducting a longer-term follow-up study could help determine if there are any lasting effects of primary PCI on mental health.
3. Compare primary PCI to alternative therapies: It would be interesting to compare the impact of primary PCI on mental health to those who undergo alternative therapies, such as thrombolytic therapy or medical management. This type of research could help determine if those who undergo primary PCI are better off in terms of mental health outcomes compared to other treatment methods.
4. Investigating the impact of anxiety and depression on long-term outcomes following primary PCI: While our study examined the impact of primary PCI on anxiety and depression, future studies could address the reverse relationship by exploring how anxiety and depression might affect long-term outcomes following primary PCI, such as rates of re-hospitalization or mortality.
5. Investigating the impact of specific cardiac conditions on anxiety and depression following primary PCI: Different cardiac conditions may have different effects on mental health outcomes following primary PCI. Examining the specific impact of conditions such as acute myocardial infarction, arrhythmias or heart failure on anxiety and depression could provide valuable insights into how best to support patients with these conditions.
6. Investigating the impact of mental health status on secondary prevention measures: We can examine whether patients with poor mental health are less likely to adhere to lifestyle changes such as exercise and diet or medication regimes that could prevent further cardiac events.
7. Comparing different methods of treatment for anxiety and depression following primary PCI: Patients who undergo primary PCI may receive different methods of treatment for anxiety and depression, including medications, psychotherapy, or referrals for additional mental health services. Comparing these treatment methods and their effectiveness would be an important next step in determining how best to address these mental health issues in patients who have undergone primary PCI.

These are just a few areas for potential future studies related to primary PCI and anxiety and depression. Our article emphasizes the importance of further research in this area to improve the care and outcomes of patients who have undergone primary PCI.

Overall, there are many avenues for future studies to build upon our findings, and explore the psychological impacts of primary PCI in greater detail.

## 6. Conclusions

Given the strong link between depression and anxiety and acute myocardial infarction, there is a growing need to improve screening, diagnosis, and management of psychiatric disorders in this patient population. The management of psychiatric disorder after myocardial infarction requires a collaborative and integrative approach, involving cardiology, psychiatry, and primary care.

Our study highlights the importance of treating psychiatric disorders in this population as it can worsen outcomes and lead to poorer quality of life. This study provides evidence that depression and anxiety are common issues in acute myocardial infarction (AMI) survivors and interventions addressing the condition should be a routine part of care. The study underscores the need for healthcare providers to be aware of the increased risk of mental disorders in individuals who have suffered from AMI, and should actively screen and manage psychiatric disorders in this patient population.

Accurately identifying and treating psychiatric disorders in patients following an acute myocardial infarction is essential for improving their overall health outcomes and quality of life. Patients with depression and anxiety, following an acute myocardial infarction, should be screened and referred for appropriate interventions, including psychological counseling, pharmacotherapy, and behavioral interventions. Early detections and timely treatment can help alleviate psychiatric symptoms and improve overall patient outcomes and improved quality of life.

## Figures and Tables

**Table 1:** The main socio-demographic characteristics of the sample.

	Socio-Demographic			Medical History			Risk Factors	
	N	%		N	%		N	%
Gender			HTA			Smoking		
Male	65	73.9	Present	70	79.5	Yes	17	19.3
Female	23	26.1	Absent	18	20.5	No	71	80.7
Living place			Diabetes melitus			Alcohol		
Urban	48	54.5	Present	36	40.9	Yes	28	31.8
Rural	40	45.5	Absent	52	59.1	No	60	68.2
Age						Hypercholesterolemia		
<60 years	16	18.2				Yes	21	23.9
60–70 years	47	53.4				No	67	76.1
>70 years	25	28.4						

**Table 2:** Mean Values of Hamilton Score for Depression.

	Before	1 Month	6 Months	12 Months
HAM-D17	7.59 ± 2.5	5.32 ± 2.68	4.06 ± 2.45	4.35 ± 2.54
Gender				
Female	8.78 ± 2.04	6.65 ± 2.53	5.17 ± 2.5	5.7 ± 1.96
Male	7.17 ± 2.53	4.85 ± 2.59	3.66 ± 2.32	3.88 ± 2.56
Age				
<60 years	6.50 ± 1.93	4.19 ± 1.76	2.94 ± 1.48	3.38 ± 1.54
60–70 years	7.11 ± 2.56	4.43 ± 2.19	3.23 ± 1.84	3.36 ± 1.71
>70 years	9.2 ± 1.98	7.72 ± 2.54	6.32 ± 2.51	6.84 ± 2.59
Hypertension				
Present	7.73± 2.42	5.47 ± 2.67	4.13 ± 2.47	4.38 ± 2.52
Absent	6.86 ± 2.78	4.72 ± 2.7	3.77 ± 2.39	4.22 ± 2.65
Diabetes mellitus				
Present	7.33 ± 2.38	4.86 ± 2.3	4.03 ± 2.54	4.22 ± 2.38
Absent	7.77 ± 2.59	5.65 ± 4.08	4.08 ± 2.41	4.44 ± 2.66

HAM-D17: Hamilton Depression Rating Scale-17.

**Table 3:** Depression Level Across Time Intervals.

Depresion Level (N, %)	Before	1 Month	6 Months	12 Months
Normal	42 (47.7%)	72 (81.8%)	77 (87.5%)	78 (88.6%)
Slight	46 (52.3%)	16 (18.2%)	11 (12.5%)	10 (11.4%)
Risk *		0.35	0.22	0.24
CI 95%		0.22–0.57	0.12–0.41	0.13–0.43
*p*		<0.001	<0.001	<0.001

* Risk of depresion comparing with preintervention level.

**Table 4:** Anxiety Level Comparison Across Time Intervals.

Anxiety Level (N, %)	Before	1 Month	6 Months	12 Months
Normal	12 (13.6%)	42 (47.7%)	68 (77.3%)	64 (72.7%)
Slight	66 (75%)	44 (50%)	20 (22.7%)	23 (26.1%)
Moderate	10 (11.4%)	2 (2.3%)	0	1 (1.1%)
Risk *		0.61	0.26	0.32
CI 95%		0.49–0.75	0.18–0.39	0.22–0.44
*p*		<0.001	<0.001	<0.001

* Risk of anxiety comparing with preintervention level.

**Table 5:** Longitudinal Association between HAM-A Scores and Medical History Factors.

	Before	1 Month	6 Months	12 Months	*p*
HAM-A	7.23 ± 2.66	4.67 ± 2.38	2.99 ± 1.97	3.55 ± 2.48	<0.001
Gender					
Female	9.22 ± 2.5	6.52 ± 2.43	4.26 ± 1.63	5.17 ± 2.42	<0.001
Male	6.52 ± 2.35	4.02 ± 1.99	2.54 ± 1.89	2.97 ± 2.25	<0.001
Age					
<60 years	5.75 ± 2.41	2.63 ± 1.54	1.13 ± 1.2	0.81 ± 1.05	<0.001
60–70 years	6.51 ± 2.22	4.17 ± 1.81	2.62 ± 1.38	3.13 ± 1.36	<0.001
>70 years	9.52 ± 2.16	6.92 ± 2.04	4.83 ± 1.79	6.08 ± 2.47	<0.001
Hipertension					
Present	7.21 ± 2.76	4.8 ± 2.42	3.14 ± 1.98	3.83 ± 2.42	
Absent	7.28 ± 2.29	4.17 ± 2.17	2.39 ± 1.85	2.61 ± 2.12	
Diabetus mellitus					
Present	6.69 ± 2.74	4.3 ± 2.35	2.86 ± 1.66	3.58 ± 2.28	
Absent	7.59 ± 2.56	4.92 ± 2.38	3.07 ± 2.16	3.51 ± 2.63	

HAM-A: Hamilton Anxiety Scale.

## Data Availability

Not applicable.
